# Patients’ and clinicians’ perspectives on item importance, scoring, and clinically meaningful differences for the Endometriosis Symptom Diary (ESD) and Endometriosis Impact Scale (EIS)

**DOI:** 10.1186/s12955-020-01579-7

**Published:** 2021-01-06

**Authors:** Helen Kitchen, Christian Seitz, Andrew Trigg, Natalie Aldhouse, Thomas Willgoss, Heinz Schmitz, Adam Gater, Christoph Gerlinger, Claudia Haberland

**Affiliations:** 1 DRG Abacus, The Lexicon, Mount Street, Manchester, M2 5NT UK; 2 Bayer AG, Müllerstraße 178, 13353 Berlin, Germany; 3 Adelphi Values, Adelphi Mill, Grimshaw Lane, Macclesfield, SK10 5JB UK; 4 Department of Gynecology, Obstetrics and Reproductive Medicine, University Medical School of Saarland, 66421 Homburg/Saar, Germany

**Keywords:** Endometriosis, Endometriosis Symptom Diary (ESD), Endometriosis Impact Scale (EIS), Women’s health, Patient-reported outcome measures, Patient preference, Qualitative research, Clinically important difference (CID)

## Abstract

**Background:**

The Endometriosis Symptom Diary (ESD) and Endometriosis Impact Scale (EIS) are patient-reported outcome measures developed to evaluate efficacy in clinical trials and clinical practice. The ESD is a daily electronic diary assessing symptom severity; the EIS is a weekly electronic diary assessing symptom impact. This study explored the importance of symptoms (ESD items) and impacts (EIS domains), perspectives on scoring algorithms, and clinically important difference (CID) thresholds to inform clinical trial score interpretation.

**Methods:**

Endometriosis patients in Germany (n = 8) and the US (n = 17), and expert clinicians (n = 4) in Germany, the US, Spain, and Finland participated in semi-structured qualitative interviews comprising structured tasks. Interview transcripts were analyzed using thematic analysis techniques.

**Results:**

Quality and severity of endometriosis-associated pelvic pain varied considerably among patients; some experienced pelvic pain daily, others during menstrual bleeding (dysmenorrhea) only. Patients and clinicians ranked “worst pelvic pain” as the most meaningful pain concept assessed by the ESD, followed by constant and short-term pelvic pain. Preferences for summarizing daily pain scores over the 28-day menstrual cycle depended on individuals’ experience of pain: patients experiencing pain daily preferred scores summarizing data for all 28 days; patients primarily experiencing pain during selected days, and their treating clinicians preferred scores based on the most severe pain days. Initial CID exploration for the “worst pelvic pain” 0–10 numerical rating scale (0–10 NRS) revealed that, for most patients, a 2- or 3-point reduction was considered meaningful, depending on baseline severity. Patients and clinicians ranked “emotional well-being” and “limitations in physical activities” as the most important EIS domains.

**Conclusions:**

This study informs the use of the ESD and EIS as clinically relevant measures of endometriosis symptoms and their impact. Findings from the ESD highlight the importance of individual-patient assessment of pain experience and identify “worst pelvic pain” as the most meaningful symptom assessed. Aggregating scores over the 28-day menstrual cycle may inform meaningful endpoints for clinical trials. Diverse EIS concepts (e.g. impact on emotional well-being and physical activities) are meaningful to patients and clinicians, emphasizing the importance of evaluating the impact on both to comprehensively assess treatment efficacy and decisions.

***Trial registration*:**

Not applicable. Qualitative, non-interventional study; registration not required.

## Background

Endometriosis is an estrogen-dependent disorder characterized by the presence of endometrial-like tissue outside the uterus, which induces a chronic inflammatory reaction [1, 2]. It affects approximately 10–15% of women, although the exact prevalence is unknown due to diagnostic delay, increasing to 30–50% among those with infertility [3–5]. Pelvic pain is the main symptom associated with endometriosis, which can present during vaginal bleeding (dysmenorrhea), during sexual intercourse (dyspareunia), or independent of vaginal bleeding (non-menstrual pelvic pain) [6]. Patients can also experience lower back pain or abdominal discomfort [6]. Endometriosis symptoms can affect a patient’s physical, mental, and social well-being, impairing quality of life [1, 7]. Endometriosis is a chronic disorder and although medical and surgical treatments can alleviate symptoms, 5–59% of women continue to experience pain following treatment, 11–19% of women experience no pain relief at all, and 17–34% experience recurrence of pain symptoms after treatment cessation [8–10].

In clinical trials, treatment effect is generally assessed by investigating change in endometriosis associated pelvic pain (EAPP) such as non-menstrual pelvic pain, dysmenorrhea, and dyspareunia [11, 12]. Considering the inherently subjective nature of these symptoms, and in the absence of biomarkers, they can only be rated appropriately by patients’ self-report using patient-reported outcome (PRO) measures. A systematic review of EAPP assessment has suggested that a visual analogue scale (VAS) or a numeric rating scale (NRS) adapted for endometriosis may optimize the assessment of EAPP in clinical research and practice and identified a potential gap in availability of measures developed on the basis of patient input [13]. Over the past few years several PRO measures have been developed for use to assess key clinical trial endpoints in the evaluation of endometriosis treatments addressing this gap, including the Endometriosis Symptom Diary [14] and the Endometriosis Pain Daily Diary (EPDD) [15]; both measures assessing similar concepts but with notable differences as described previously [14]. In conjunction measures have also been developed to assess the impact of endometriosis pain including the Endometriosis Daily Pain Impact Diary [16] and the Endometriosis Impact Scale (EIS) [14].

The measures of interest in this study, the ESD and EIS, were developed in accordance with scientific standards including FDA Guidance for Industry [17] and the European Medicines Agency (EMA) reflection paper [18]. The content of the ESD and EIS was developed based on a review of published literature, concept elicitation interviews with patients in the US, Germany, and France (n = 45), cognitive interviews with patients in the US and Germany (n = 31), and consultations with PRO and clinical experts throughout the development [14].

The ESD is a daily electronic diary which assesses the experience of endometriosis symptoms over the past 24 h mostly reported on a 0–10 NRS. The EIS is a weekly electronic diary comprising items assessing the impact of endometriosis symptoms on patients’ daily lives over the past 7 days using a 5-point VRS [14, 19]. The psychometric properties of the ESD and the EIS were further investigated in accordance with scientific standards in the Validation study for Endometriosis PRO (VALEPRO), a prospective, non-interventional study conducted in the US and Germany which supported the ESD and EIS scores as reliable, valid, and sensitive to change for investigating endpoints in endometriosis clinical studies [19, 20]. All validation work was conducted under consultation with health authorities. Both PROs were included in a phase IIb trial to further confirm their measurement properties under clinical trial conditions [21].

As a daily-completed, multi-item measure, patients’ daily responses to individual ESD items can be aggregated using multiple different scoring approaches. For example, data can be combined using scores either from every day or from selected days according to the relevance to the concept of interest, such as average pain or worst pain and summarized as either frequency, mean, range. For Patient Focused Drug Development, the priority of these endpoints for hierarchical testing should be patient informed [22, 23]. The cyclical nature of endometriosis and its symptomatic heterogeneity can contribute to significant variation in patient experience and perception of symptom impact, making careful consideration of endpoints defined by diaries essential [22]. There is limited available guidance on developing and translating daily assessments into meaningful, sensitive endpoints. In addition, to interpret these endpoints, a threshold for meaningful within patient change must be determined [23]. Quantitative approaches (including anchor based methods) remain the standard in identifying CID thresholds, however, qualitative methods have gained traction [24], and the importance of collecting patient insight into the concept of meaningful change is increasingly recognized [25]. Especially, qualitative input from patients living with endometriosis, alongside clinical insight, can inform data interpretation in a way that will be meaningful and resonate with patients [22].

This study sought to generate additional insights from patients with endometriosis and treating clinicians to inform the implementation of the ESD and the EIS in endometriosis clinical trials, especially informing clinical endpoint selection and score interpretation. The specific objectives of this study were to understand: (1) the ESD items and EIS domains that would inform the most meaningful clinical trial endpoints; (2) preferred scoring algorithms for aggregation of the daily responses of the ESD “worst pelvic pain” item; and (3) clinician and patient perspectives of meaningful change in the ESD “worst pelvic pain” score.

## Methods

This was a multinational qualitative interview study conducted with patients with endometriosis and clinical experts treating patients with endometriosis. Qualitative and quantitative data were collected in the interview.

### Recruitment

Four clinicians, one each from the US, Germany, Spain and Finland, were recruited via an in-house clinician database and by contacting national endometriosis associations. Eligibility criteria required the clinicians to be in clinical practice and regularly treating ≥ 5 patients with endometriosis per month. No formal ethical approval was required for the clinician interviews, but all participants provided written informed consent.

Patients were recruited from three clinical sites in the US and one clinical site in Germany through referral from clinicians participating in the ESPARIOS clinical trial (NCT02203331). Patients were referred based on pre-defined eligibility criteria, which were designed to allow comparability between the ESPARIOS study population and future endometriosis clinical trial populations. Patients were eligible for inclusion if they were a premenopausal female ≥ 18 years of age with their endometriosis diagnosis confirmed by laparoscopy or laparotomy within 10 years of the interview, and had experienced EAPP within 4 weeks of the interview. All patients provided informed consent and ethical approval of the protocol was received (New England IRB #16-062; Meridian Health IRB# #201701052J; Baden Württemberg ethics committee #F-2016-072).

### Interview process

Interviews were conducted in line with accepted industry best practice [25–28]. Clinician interviews were conducted via telephone in the clinician’s native language, and lasted 45–90 min (median 57 min, mean 64 min) no more than 60 min; patient interviews were conducted face-to-face by a trained, female qualitative investigator in the participant’s native language and lasted between 45–105 min (median 66 min, mean 67.8 min). The ESD and EIS had been previously translated and linguistically validated in native endometriosis patients from US English to Spanish, German, and Finnish using industry standard methods [29, 30]. Separate semi-structured interview guides were developed for the clinician and patient interviews, and comprised a brief exploration of patients’ lived experiences of endometriosis, followed by four structured cognitive exploration tasks in line with the study objectives.

### Cognitive exploration tasks

#### ESD item and EIS domain importance

To explore ESD item/EIS domain importance, clinicians were asked to allocate 100 points across each measurement concept or domain in a constant sum scaling task. Clinicians provided two distributions, one to represent the clinician perspective, and a second to represent their perception of the patient perspective (Example in Additional file 1: Additional Fig. 1). In comparison, patients were asked to rank the ESD items and EIS domains (Additional file 1: Additional Fig. 2) in order of relative importance from the patient’s own perspective. Patients were permitted to give two or more items/domains the same importance ranking if they wished. Responses from both clinicians and patients were used as a prompt for further discussion around the relative importance of the discussed ESD items/EIS domains.

#### Scoring algorithms for the ESD “worst pelvic pain” item

Clinician and patient insights were collected on appropriate algorithms for aggregation of the daily responses on the ESD “Worst overall pelvic pain” 0–10 NRS using four calendar depictions of potential scores to explore which was most meaningful (Additional file 1: Additional Fig. 3): mean score of the 7 worst days in the past 28 days (Score A), mean score of all 28 days (Score B), percentage of days with worst pain ≥ 7 in the past 28 days (Score C), or percentage of days with worst pain ≥ 4 in the past 28 days (Score D). Responses from clinicians and patients were used to evaluate preferences for score aggregation for informing the assessment of patient responses during a clinical trial.

#### ESD “Worst overall pelvic pain” 0–10 NRS cut-off scores

Patients described each level of pain, with particular detail provided about the patient experience at the two pre-suggested 0–10 NRS cut-off points: 0–10 NRS 4 (moderate EAPP) and 0–10 NRS 7 (severe EAPP) [31]. This informed the appropriateness of scoring algorithms that included scores of ≥ 4 and ≥ 7 (as detailed in Score C and Score D; Additional file 1: Additional Fig. 3). In addition, clinicians and patients described the impact that “Worst overall pelvic pain” had on the patients.

Clinicians were asked, which values of the ESD “Worst overall pelvic pain” 0–10 NRS they considered to represent for “mild,” “moderate,” and “severe” pain and to discuss the clinical meaningfulness of scores > 4 and > 7. To facilitate discussion, study participants presented with a blank 0–10 NRS (Additional file 1: Additional Fig. 4) and asked to use pre-defined and/or blank labels to assign descriptions to points on the scale.

#### Meaningful change on the ESD “worst pelvic pain” 0–10 NRS

Clinicians and patients were asked to describe the score changes on the ESD “Worst overall pelvic pain” 0–10 NRS that would be considered meaningful or important in the context of receiving endometriosis treatment. To facilitate this, patients were shown the ESD “Worst overall pelvic pain” 0–10 NRS marked with various changes in score (Additional file 1: Additional Fig. 5), and asked to describe what such a change would mean to them in terms of how they would feel or function. Where possible, this discussion took place in the context of true changes patients had experienced, while clinicians described meaningful change scores both from a clinical management perspective and from the perspective of their patients.

### Data analysis

Interviews were audio recorded, transcribed verbatim, and translated to English where required. Participants were allocated a code to allow data to be reported anonymously, and interview transcripts were reviewed in full to remove all identifying information. Qualitative data were subject to thematic analysis [32, 33] facilitated by use of ATLAS.ti v7.5 software; quantitative data were explored using descriptive statistics in Microsoft® Excel.

## Results

### Study sample

Four clinicians from the US, Germany, Spain, or Finland (each n = 1) were interviewed. All worked within university hospitals and were involved in endometriosis diagnosis, treatment, and patient management. Overall, 25 patients with endometriosis from the US and Germany participated; demographic and clinical characteristics are presented in Table 1.

**Table 1:** Patient demographic and clinical characteristics

	Patients, n (%) N = 25
**Participants of the ESPARIOS study**	
Yes	8 (32)
No	17 (68)
**Mean age in years, (SD) [Range]**	33 (6) [19–47]
**Country of residence**	
USA	17 (68)
Germany	8 (32)
**Ethnicity**	
Hispanic or Latino	1 (4)
Not Hispanic or Latino	24 (96)
**Race**	
White	21 (84)
Black	4 (16)
**Education**	
University/college degree or equivalent professional qualification	14 (56)
No university/college degree or equivalent professional qualification	9 (36)
Not reported	2 (8)
**Employment status**	
Employed	21 (84)
Full-time homemaker	1 (4)
Not working due to medical reasons	2 (8)
Not reported	1 (4)
**Mean years since endometriosis first suspected by clinician (SD) [Range]**^a^	8.0 (5.8) [0.3–18.9]
**Comorbidities affecting > 1 patient, n (%)**	
Anemia	3 (13)
Depression	3 (13)
Migraine	3 (13)
Ovarian cysts	3 (13)
Hypertension	2 (8)
**Treatment received (previous or current)**^a^	
Surgery	18 (75)
Analgesics	11 (46)
Combined oral contraceptive	13 (54)

^a^Data only available for 24 participants

Some categories do not sum to N = 25 due to: Treatments received were not mutually exclusive, some patients received more than one therapy; comorbidities were not mutually exclusive, some patients had more than one comorbidity and other patients had no comorbidities

SD = standard deviation

### Patients’ experiences of pelvic pain related to endometriosis

Experiences of EAPP varied considerably between patients, with some patients experiencing pain every day and others only experiencing pain around the time of their menstrual period or during intercourse. Examples of patient pain profiles (mild, moderate/severe, and unpredictable) are shown in Table 2.

**Table 2:** Examples of three different patient pain profiles

Patient ID	Daily pain not associated with vaginal bleeding	Pain prior to/following vaginal bleeding	Pain during vaginal bleeding	Pain associated with sexual intercourse	Patient quotes when asked “Tell me how endometriosis makes you feel physically?”
US-04	Constant mild pain	No different to rest of month	Severe	No different to rest of month	“On a daily experience, it’s not that bad. You kind of get used to the pain. It’s when your menstrual cycle comes that it’s really bad”
US-09	Unpredictable: Either no pain or severe pain	No different to rest of month	No different to rest of month	No different to rest of month	“Sometimes it happens, sometimes it doesn’t.[…] It might flare up, it might not.[…] If it happens, then it happens, and I’m just kind of out of the ballgame at that point”
US-17	Constant moderate pain	Severe	Severe	Severe	“…lately it has been pretty doggone constant”

### ESD item importance

Patients and clinicians gave similar importance to each item of the ESD (Table 3). “Worst overall pelvic pain” (ESD item 1) was rated by all clinicians and most patients (n = 15/24; 63%) as the most or second-most important ESD item. Patients explained that “Worst overall pelvic pain” encompassed all of the pain types experienced in association with endometriosis, included pain during presence or absence of vaginal bleeding, and was therefore the pain that they experienced most often and had the greatest impact on their lives:

- “It summarizes all the pain.” (US-16)
- “[It’s the most important] because if I can't fix that, then I can't do my daily stuff.” (US-04)
- “[This is] most important because I feel like this is a daily issue.” (US-06)

**Table 3:** Relative importance of ESD items/concepts reported by patients and clinicians

Item/concept	Patient rankings (1–7, where 1 is the most important and 7 is the least important)								Clinician mean constant sum (0–100)
	Number of patients (N = 24^a^) attributing item to each ranking							Patient median rank	
	1	2	3	4	5	6	7		
Worst overall pelvic pain	11	4	4	2	2	0	1	2	28.75
Worst constant pelvic pain	8	6	2	2	3	3	0	2	22.5
Pain associated with bleeding^b^	2	4	3	4	4	4	3	4	10
Worst short-term pelvic pain	5	1	5	4	3	3	3	4	13.75
Pain due to sexual intercourse^c^	4	1	3	4	4	3	1	4	11.25
Avoidance of sexual intercourse due to pain^d^	1	4	2	1	6	5	4	5	7.5
Pain medication use	2	0	5	3	2	2	10	5.5	5

^a^N = 24, as one patient had trouble understanding and thus was unable to provide meaningful responses

^b^Note that pain associated with bleeding was calculated by considering responses to ESD item 1 (“worst pelvic pain”) and rating of bleeding severity by ESD item 4

^c^N = 20, as four patients stated that they were unable to provide a ranking for this item as they did not experience pain during sex (N = 3) or were not sexually active (n = 1)

^d^N = 23, as one patient stated that she was unable to provide a ranking for this item as she was not sexually active

ESD = Endometriosis Symptom Diary

“Worst constant pelvic pain” was assigned the second highest priority overall for both clinicians and patients; one clinician described this pain type as “the most disabling for a woman.” Fourteen of 25 patients (56%) considered “Worst constant pelvic pain” as one of the top two most important pain types due it being a permanent “frustrating” presence in their lives and the type of pain most likely to interfere with their daily activities:

- “I obviously think the constant pain is [most important] because […] it’s constant pain, it’s all the time.[…] It makes me unhappy, it makes me irritable.” (US-03)
- “I can feel it every day, hour or minute. It is just always present.” (DE-03)

Other patients considered this item to be less important because they could anticipate their “Worst constant pelvic pain”, and had learned how to cope with it:

- “Well, the constant I can deal with ‘cause I know it’s going to be there.” (US-11)
  “Worst short-term pelvic pain” received mixed views from patients and clinicians. Some patients ranked “Worst short-term pelvic pain” with high importance due to the severity and unpredictability of this pain type. Others considered the short-lived nature of the pain comparatively easier to cope with and thus of lesser importance:
- “I have a pretty good pain tolerance. So, I know if I can just breathe through it for a minute, it goes away.” (US-04)
- “This is the worst thing for me.[…] It is the most limiting for me, the fact that it is random or unpredictable, and it just shuts me off and kills any nice moments.” (DE-05)
- “I may be sitting at my desk at work when all of a sudden the stabbing pain kicks in and I pinch my fingers on my desk and curl up with pain.” (DE-07)

There was some variability between the importance that patients assigned to the “Pain associated with bleeding” concept and “Pain during sexual intercourse” item. Patients who did not experience pain during sexual intercourse or did not experience differences in pain during bleeding days compared with non-bleeding days ranked these items of a comparably lower importance (Table 2). Clinician ratings were also comparably lower to overall worst pelvic pain, constant and short-term pain. For other patients, however, pain associated with bleeding or sexual intercourse were rated to be of highest importance. In addition, several patients found the “Pain during sexual intercourse” item important due to its impact not just on themselves, but also their partners.

- “[Bleeding days] are the days where I am in the most pain and the most incapacitated.” (US-03)
- “I put ‘Pain associated with bleeding’ [as rank] 7 because for me I never really had pain with bleeding, so that to me is not a factor.” (US-01)
- “I think [‘Pain due to sexual intercourse’] is just very important to me because that's something that I would like to give [my husband].[…] It's frustrating because I can't give him that one particular thing that he really desires as much as he would like.” (US-02).

Patients and clinicians interviewed both ranked “Avoidance of sexual intercourse” and “Pain medication usage” as ESD items of minor importance to them. However, some patients rated “Pain medication usage” highly due to concerns related to addiction or side effects.

### EIS domain importance

Clinicians mostly agreed with patients on the relative importance of the different EIS concepts (Table 4). Patients and clinicians ranked “Negative impact on your emotional well-being” to be of high importance, as their emotional response to EAPP, e.g. feeling tearful, irritable and angry, had a substantial impact on their ability to undertake any daily activity or to socialize.

- “When I don’t feel good, I am in a bad mood. It’s easy for me to be irritable or stressed out. And so, that affects my family and it affects work.” (US-13)
- “When I'm not in a good mood I don't want to be around people. I'm a social butterfly so people don't understand when I don't want to be around them because I'm in pain.” (US-02)

**Table 4:** Relative importance of EIS concepts reported by patients and clinicians

Item/domain	Number of patient (N = 24^a^) ranking item/domain at each value, where 1 is the most important and 4 is the least important				Patient median (ranked 1–4)	Clinician mean (constant sum 0–100)
	1	2	3	4		
Negative impact on your emotional well-being	11	5	6	2	2	27.5
Limitation in your physical activities	10	6	6	2	2	35
Other limitations	3	9	6	6	2.5	22.5
Limitation in your sexual activities	5	3	4	10	3	15

^a^N = 24, as one patient had trouble understanding and thus was unable to provide meaningful responses

EIS = Endometriosis Impact Scale

“Limitations in your physical activities” also ranked highly in the patients’ ratings:, suggesting, if they could not move, they could not work, socialize, or take part in leisure activities.

- “Ok. My first one I put as most important was physical activities, which is my exercise, the exercise, like six days a week, so for me, even if I’m all day in my job. So for me that is the most important, because if I don’t exercise, and if I take out two days of exercise, I get crabby, it’s like I just need that stimulation or something.” (US-01)
- “I want to be able to go out and play with my son without needing to come in and sit down. I want to be able to sit through a movie […] I will have to change positions five hundred thousand times because it gets uncomfortable.” (US-03)

This ranking was echoed by clinicians. One clinician noted that “one thing is a consequence of the other,” and another clinician stated that if patients can go about their physical activities, they have “fewer emotional problems”.

The EIS “Other limitations” domain includes items assessing social and leisure activities, paid work or study, household activities, difficulty concentrating, and/or difficulty sleeping. Of these items, patients considered difficulty sleeping and an inability to work or socialize as important items.

- “I want to be able to work continuously and not have to miss shifts and miss out on money. I do still have bills. But when it happens I have no choice but to leave.” (US-11)
- “Sleeping; oh my goodness, yeah. I've been asleep and it's like a sharp—it hits me, it wakes me up out of my sleep. So, yeah, that's—it's not fun.” (US-05)

Overall, patients and clinicians considered “Limitation in sexual activities” as comparably less important to other domains. Clinicians described “Limitation in sexual activities” as not of high importance to some patients. However, for some patients this was a very important concern due to the impact on their partner as well as themselves.

- “That would make me happier, number one it would make my husband happier, make my family happier […] because when you have two happy parents you would be happier.” (US-03)
- “It’s not that it’s not important, but everything else came prior.” (US-16)

### Scoring algorithms for the ESD “worst pelvic pain” 0–10 NRS

Overall, patients expressed mixed preferences regarding the four different scoring algorithms proposed for the “Worst overall pelvic pain” 0–10 NRS for aggregating the daily scores over a 28 day period. Individual preferences seemed dependent on the patient’s own pain profile. Patients’ and clinicians’ perceived advantages and disadvantages of each algorithm are presented in Table 5.

**Table 5:** Comparison of four algorithms for aggregation of daily responses for ESD “worst pelvic pain” NRS

	Meaningful	Not meaningful
Score A: average of the worst 7 days	Patients, 36%/clinicians, 75% Meaningful for patients with significant pain for one week of the month and lower pain for the remaining days Meaningful for patients that would like treatment to address the “most severe” pain days “There is like just an always—I guess an always constant something [low level pain], but I can deal with that… So, I guess—I mean it doesn’t bother me. I would rather look at that target like week or ten days or something like that.” (US-12)	Patients, 44%/clinicians, 25% Not meaningful for patients with significant pain lasting ≥ 7 days a month Not meaningful for patients who consider pain of lesser severity to also be important “Just looking at the seven worst days … if someone has very bad pain for one and a half weeks in a row, then this calculation makes no sense at all” (DE-04)
Score B: average of all the days	Patients, 40%/Clinicians, 25% Meaningful for patients with pain every day of the month Meaningful for patients who consider pain of any severity to be important “I mean, you know, yes, those seven [worst] days, that sucks when you are hurting that bad.[…] But it’s, like I said… I could deal with seven days. I can handle that. It’s the 28 that gets so hard.” (US-03)	Patients, 48%/Clinicians, 75% Not meaningful for patients with pain-free or “mild” pain days as these would “dilute” the score, causing some patients’ conditions to appear less severe Not meaningful for patients that would like treatment to address the “most severe” pain days “I think it would downplay how severe the pain is with endometriosis if you had somebody that the other four weeks were cool and tolerable and really didn't have to take much, but that first week was horrible.” (US-04)
Score C: percentage of days with pain ≥ 7	Patients, 28%/clinicians, 50% Meaningful for patients with significant pain for > 7 days a month and lower pain for the remaining days Meaningful for patients that would like treatment to address the “most severe” pain days “I think that’s a fair way to look at the data because if you’re scoring a seven or higher, it’s affecting your life. You’re taking medication or you can’t work or do the things that you’re supposed to be doing … So, I think saying, “Oh, 29% of the month she really had a problem,” I think that is fair.” (US-16)	Patients, 56%/clinicians, 50% Not meaningful for patients who would also like pain scores of < 7 to be addressed by treatment A cut-off score of 7 is too high to identify the “most painful” days for some patients Requires all patients to use the same definition of a pain score of 7 “It needs to be, you know, more than just those that are over 7, because a 5′s bad, you know, a 5 is a lot of pain and especially in that area because it affects your whole life. This area right here affects your standing, your sitting, your, oh, you know, it affects sex, it affects everything, so…” (US-03)
Score D: percentage of days with pain ≥ 4	Patients, 60%/clinicians, 50% Meaningful for patients that would like treatment to address all of the days they need at least some help to manage the pain “…If I’m at a 7, that’s going to be bad, but even at a 4 or 5 my mood is different, my demeanor is different. I’m not getting up and […] fixing my hair, you know. My hair is going in a messy band and I’m going to put my pajama pants on. The 4 and above affects your life more than just a seven or above.” (US-03)	Patients, 32%/clinicians, 50% Not meaningful for patients that consider any pain to meaningful; this method would not capture days with pain scores 1–3 “…I think that paying attention to any pain at any level is important.” (US-02)

ESD = Endometriosis Symptom Diary; NRS = numerical rating scale. Note that patient ratings do not total 100% because some patient responses could not be clearly categorized into meaningful vs non-meaningful

### ESD “Worst overall pelvic pain” 0–10 NRS cut-off scores

Patients and clinicians described “mild”, “moderate”, and “severe” pain related to the

**Fig. 1:**
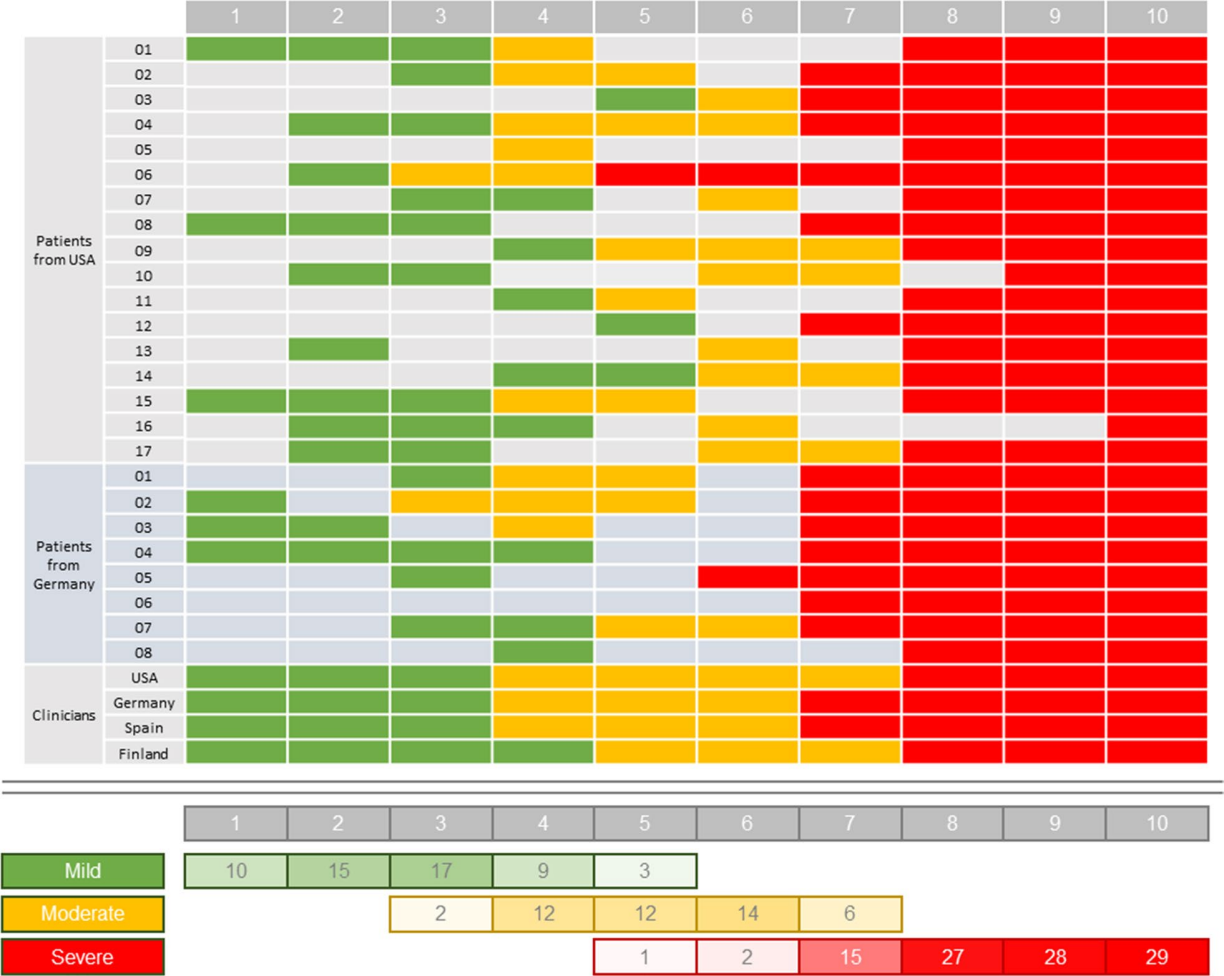
Patient and clinician uses of “mild,” “moderate,” and “severe” with the ESD NRS. Green cells indicate application of “mild” descriptor to NRS value, yellow cells indicate application of “moderate” descriptor to NRS value, red cells indicate application of “severe” descriptor to NRS value, and gray cells indicate no descriptor was allocated to the NRS value. Total scores at the bottom of the figure indicate the number of patients allocating each label to each NRS value. ESD = Endometriosis Symptom Diary; NRS = numerical rating scale

0–10 NRS. In general patients and clinicians reported the mild/moderate boundary to be at approximately 4 and the moderate/severe boundary to be at approximately 7 on the 0–10 NRS (Fig. 1).

Scores < 4 were considered to be mild and not impairing patients’ lives, whereas limitations became more apparent at a score of 4 on the 0–10 NRS. Clinicians suspected that patients would seek treatment at this point; indeed, many patients reported the use of non-steroidal anti-inflammatory drugs at scores > 4. Most patients reported “noticing” the pain at this level, although the majority did not consider themselves to be functionally impaired, commenting that “you can still do what you need to do, but you don’t feel good doing it” (US-03). Some patients noted that as they were conscious of pain, their mood would be affected.

In general, clinician perceptions of scores of 7 on the 0–10 NRS were more severe than those provided by patients; clinicians considered scores of 7 on the 0–10 NRS to be severely limiting and to require immediate medical attention. Patients, however, reported that this level of pain was just “the start of a peak” (US-15) and would subsequently get considerably worse. Some patients reported considerable physical limitations at a score of 7 on the 0–10 NRS and described reducing their activities to only those that were most necessary. Scores of 7 were also described as a turning point in patients’ moods, with patients feeling miserable and withdrawn. A summary of patient descriptions of each point on the 0–10 NRS is presented in Table 6.

**Table 6:** Patients’ descriptors used for the ESD “worst pelvic pain” 0–10 NRS

NRS	Patients’ descriptors for each pain level of the ESD “worst pelvic pain” 0–10 NRS
0	No pain
1	The pain is barely noticeable, patients are functional, able to work, and not taking medication
2	The pain is still mostly barely noticeable and described as “mild.” A minority of patients experience feelings of irritation
3	The pain is becoming more noticeable and a little bothersome—the majority of patients continue to describe it as “mild” but for some it is becoming more “moderate.” Patients are beginning to experience slight limitations in their functionality
4	The pain is now largely described as “moderate.” Function is slightly limited for many patients, but patients generally remain active, albeit feeling frustrated and irritable due to the increasing pain. Many patients begin taking mild medication –mostly NSAIDs
5	“Mild,” “moderate,” and “severe” are all descriptors used by participants to describe pain at NRS = 5, although the majority continue to consider this a “moderate” level pain. In addition, patients begin to experience slight emotional distress and/or begin the use of mild medication. No changes in functionality are reported
6	Patients are now less functional and reducing their workload; putting off chores that don’t need to be completed imminently. Patients continue to attend work, but may then dedicate their evenings to rest. A minority of patients begin to use stronger pain medication—for example, narcotics
7	Patients largely describe NRS = 7 as severe pain. Patients are doing only the “bare minimum” of daily chores, and a minority of patients do not attend work. Many patients begin to use stronger pain medication
8	All patients describe the pain as “severe.” Many patients do not attend work, and of those that continue to attend, they are reducing their duties. More patients still begin to use stronger pain medication
9	Pain continues to increase in severity. Patients are physically incapacitated
10	The pain is so severe that many patients find they need to go to hospital. The pain may be so agonizing that patients are vomiting. Patients describe feeling like they are dying

ESD = Endometriosis Symptom Diary; NRS = numerical rating scale; NSAID = non-steroidal anti-inflammatory drug

### Meaningful change on the ESD “Worst overall pelvic pain” 0–10 NRS

Clinicians described the minimum score change on the 0–10 NRS that would be considered meaningful following treatment for endometriosis to be dependent on the baseline score, as “it all depends on what level you are coming from” (Clin-GER) (Table 7).

**Table 7:** Patient feedback on meaningful change on the ESD “Worst pelvic pain” 0–10 NRS

Score change	Meaningful score change					Median score change desired
	− 1	− 2	− 3	− 4	− 5	
Smallest change from 8	2	7	9	6	1	3
Smallest change from 5	3	16	6	0	0	2
Smallest change from 3	15	3	7	–	–	1

ESD = Endometriosis Symptom Diary

Patient ratings supported this statement. When discussing meaningful change from a score of 8 on the 0–10 NRS, most patients perceived a 3-point reduction as being meaningful

- “On level 8, I cannot do anything, but on level 5, I could do almost everything although I would constantly feel right on the borderline to taking pain medication. I imagine that being on level 5, I might be free of pain at some points by taking pain medication.” (DE-06)
  When discussing meaningful change from a score of 5 on the 0–10 NRS, most patients described a 2-point reduction as being meaningful. One patient described a change in score from 5 to 3 as “life-changing” (US-03) and another commented that “even though it might not be fun, I could go to work normally and lead a regular social life” (DE-04).
  When discussing meaningful change from a score of 3 on the 0–10 NRS, some patients commented on the limited opportunity for meaningful change a score of 3 on the 0–10 NRS was already considered representative of “mild” pain. Other patients concluded that if their pain were “only” at a score of 3 on the 0–10 NRS, treatment would need to remove pain entirely in order to make a meaningful difference.
- “…I mean if I was at a 3 every day I’d be great.[…] A 3 I wouldn’t even notice.[…] Any change would be fine…” (US-01)
- “Well I guess level 2 would be fine… but really if it were me personally, I wouldn’t go on a 12-week treatment if I were on pain level 3. I feel that the effort I would put into that treatment would be more than the difference between levels 0 and 3." (DE-08)

## Discussion

The findings from this study supplement prior validation studies of the ESD and EIS [14, 19, 20] as fit-for-purpose measures of endometriosis symptoms and related impacts in clinical trials.

Participating patients had varied experiences of EAPP, including dysmenorrhea and dyspareunia, in line with previous findings [13, 15], and indeed our study found that some pain types, such as constant pain, short-term pain, pain associated with bleeding, or pain during sexual intercourse, were not experienced by all of the women interviewed.

Patients and clinicians prioritized “Worst overall pelvic pain” as an important measurement concept, regardless of whether it was associated with menstrual bleeding, with sexual intercourse, or neither. This finding is comparable with qualitative studies in other endometriosis patient populations that have identified both non-menstrual pelvic pain and pain during menstruation as important symptoms of endometriosis for a treatment to address [15, 34]. Therefore, a score assessing all aspects of pain related to endometriosis, such as the ESD “Worst overall pelvic pain” 0–10 NRS might be meaningful to support a key efficacy endpoint in endometriosis clinical trials.

When assessing the impact of EAPP, in line with previous findings, [15, 16] the domains “Negative impact on your emotional well-being” and “Limitations in physical activities” were considered most meaningful to patients and clinicians. Clinicians in particular emphasized the degree of association between these two different concepts and the importance of evaluating and reporting changes in the impact on both physical activity limitations and emotional well-being to inform the selection of treatment options.

Overall, all scoring algorithms used to aggregate the daily ratings over the 28-day menstrual cycle were found to have potential advantages. In addition, some general preferences by clinicians for selecting a subset of days within the 28-day cycle was observed in concordance with an understanding that pain levels frequently vary during the menstrual cycle and that days where the most severe pain was experienced might be most important to women with endometriosis. Patients did not identify a single “best” scoring option: those who experienced pain daily tended to prefer an aggregation of scores from all days within the 28-day cycle; those whose pain did not occur daily or whose pain varied in severity throughout the month generally preferred a score that included only the most severe days. Definitions of those “most severe pain days” were explored to understand the appropriateness of proposed scoring algorithms. Most patients considered a “severe” level of pain as ≥ 7″ on the 0–10 NRS, consistent with findings in the literature [13]. Patient opinions were mixed with regards to whether scores ≥ 4 or scores ≥ 7 were most important for a treatment to address. This result is in line with previous findings that “mild” “moderate” and “severe” verbal pain categories may correspond to different VAS and NRS values in the same patient on different occasions [35, 36].

The final objective of this study was to explore the changes in “worst pelvic pain” scores that patients understand as clinically meaningful (i.e., resulting in a meaningful improvement in how they feel or function). In general, patients and clinicians identified a greater degree of improvement as meaningful for higher baseline pain levels than for lower baseline pain levels. Slightly greater than the minimal clinically important difference of 10 mm on the 100 mm VAS for assessing endometriosis-associated pelvic pain presented by Gerlinger et al. [37], a 2-point change on the ESD “Worst overall pelvic pain” 0–10 NRS was mentioned by some of the study participants to capture meaningful changes. When setting a priori responder definitions, however, the expected baseline pain severity of the sample is recommended to be considered [36].

Patients frequently described the relationship between EAPP and physical limitations and the relationship between EAPP and impact on emotional well-being. These findings, and those reported elsewhere [14, 15, 19, 20, 38, 39], highlight that patients’ emotional well-being is directly influenced by pain severity and not just secondary to other aspects of endometriosis, such as avoidance of sexual intercourse and any associated feelings of guilt. Therefore, the impact of endometriosis on emotional well-being can be considered a proximal impact of EAPP. Indeed, this study found that “negative impact on emotional well-being” was considered of comparable importance as “limitation in physical activities” when assessing the most important EIS domains for measurement. This reflects the recommendations from the Initiative on Methods, Measurement, and Pain Assessment in Clinical Trials (IMMPACT), which list physical functioning and emotional functioning as core outcome domains that should be considered when designing clinical trials for chronic pain conditions [40].

As a major limitation of this study it must be noted that cognitively complex themes were discussed with patients who are not expected to have a background in this type of research. Additionally, the topic of meaningful differences on the ESD scale might have been difficult to conceptualize for any participants who had not experienced recent changes in pain. Also a difference in familiarity with the ESD and EIS existed, depending on whether patients had participated in the clinical study BAY98-7196/15832, or not; this was mitigated by all participants completing the ESD for 7 days prior to interview and the EIS once before and on the day of the interview. However, no obvious differences in findings between these two subgroups emerged. Qualitative exploration can be influenced by the researchers’ personal biases; this was mitigated by using highly experienced interviewers and the formal structure in tasks removed some elements of interpretation. Although patients and clinicians from several countries were included, the small sample sizes did not allow comprehensive cultural comparisons. Furthermore, the small sample size and the heterogeneity in endometriosis symptoms experienced means that results are not universally generalizable and should be considered in the context of other relevant qualitative and quantitative studies. An area for further exploration may be to investigate the importance of dyspareunia and limitations on sexual activities, given the polarization in importance ratings demonstrated by women in this study.

## Conclusions

The ESD and EIS provide clinically relevant assessments of endometriosis symptoms and the impact of endometriosis on patients’ lives. Insights from patients and clinicians highlight the importance of “worst pelvic pain” as measured by the ESD, and suggest that aggregating scores over the 28-day menstrual cycle can support efficacy assessments in endometriosis treatment trials. A reduction of 2–3 points on the ESD “worst pelvic pain” 0–10 NRS was considered meaningful to most patients, but patient interpretations of meaningful change were also reported as being dependent on baseline pain severity. Findings from this study highlight the importance of assessing the impact of treatments on physical limitations and emotional well-being of patients to fully understand treatment efficacy.

## Supplementary information

**Additional file 1**. Examples of completed ranking tasks and material used for cognitive exploration tasks.

## Data Availability

The datasets used and/or analyzed during the current study are available from the corresponding author on reasonable request.

## Abbreviations

B&B: Biberoglu and Behrmann
CID: clinically important difference
CPSSS: composite pelvic pain and Symptoms Scale
EAPP: endometriosis associated pelvic pain
EHP: Endometriosis Health Profile Questionnaire
EIS: Endometriosis Impact Scale
EMA: European Medicines Agency
ESD: Endometriosis Symptom Diary
ESPARIOS: Efficacy and Safety of Progestin and Aromatase Inhibitor Intravaginal Ring in endometriOSis
FDA: US Food and Drug Administration
IMMPACT: Initiative on Methods, Measurement, and Pain Assessment in Clinical Trials
IRB: Institutional Review Board
MCID: minimal clinically important difference
NRS: Numerical Rating Scale
NSAID: non-steroidal anti-inflammatory drug
PRO: patient-reported outcome
SD: standard deviation
VALEPRO: validation study for endometriosis PRO
VAS: Visual Analog Scale
VRS: Verbal Rating Scale
