# Supplementary material for: Patients’ and clinicians’ perspectives on item importance, scoring, and clinically meaningful differences for the Endometriosis Symptom Diary (ESD) and Endometriosis Impact Scale (EIS)
Source: Health Qual Life Outcomes. 2021 Jan 6;19:7. doi: 10.1186/s12955-020-01579-7 (PMC7789138; doi:10.1186/s12955-020-01579-7)

Additional files

**Additional Figure 1** Example of completed ESD (A) and EID (B) ranking tasks (Spanish clinician)


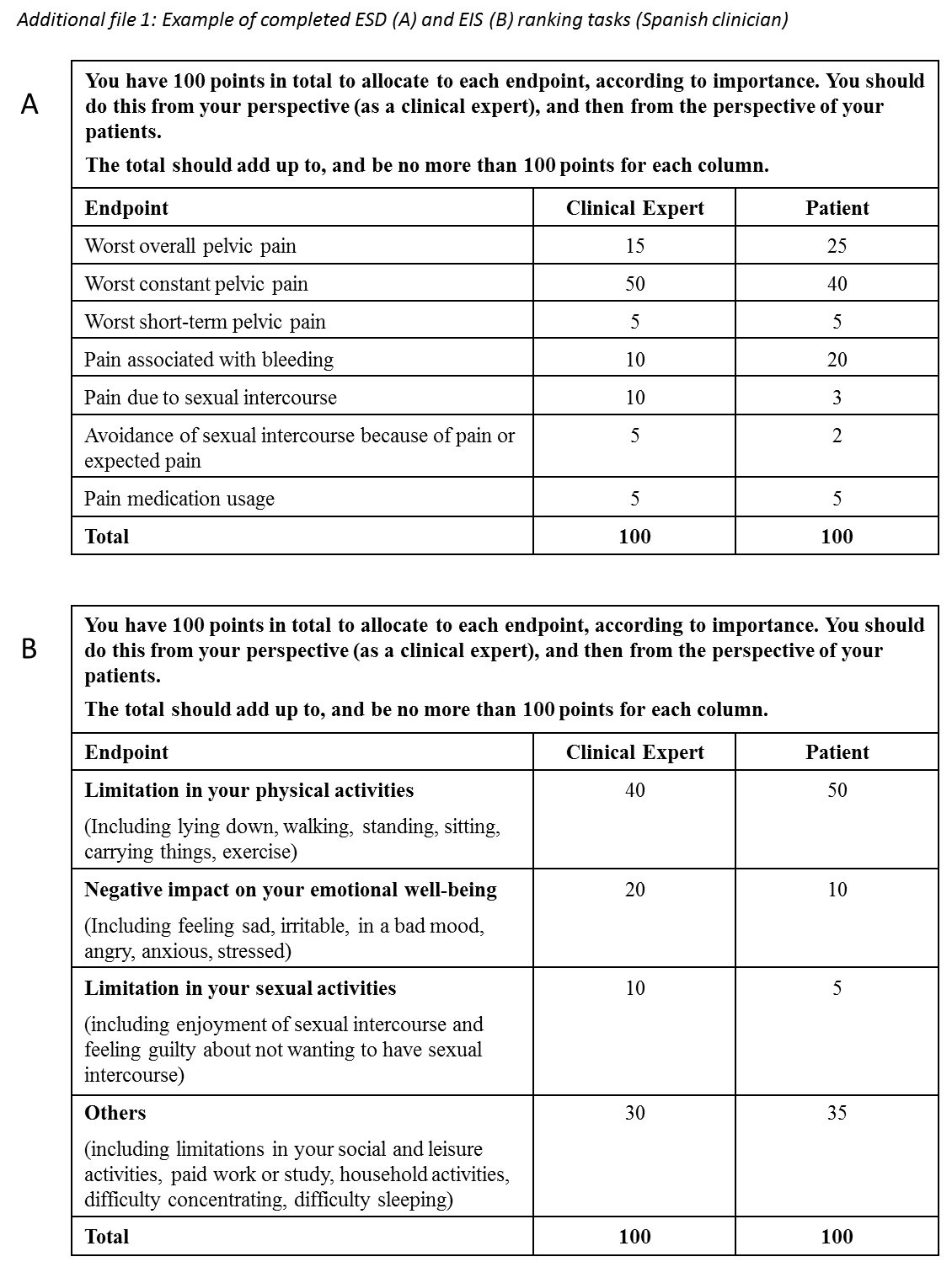


Abbreviations: *ESD* Endometriosis Symptom Diary.

**Additional Figure 2** Patient ranking activities to explore ESD item and EIS domain importance (US English version)


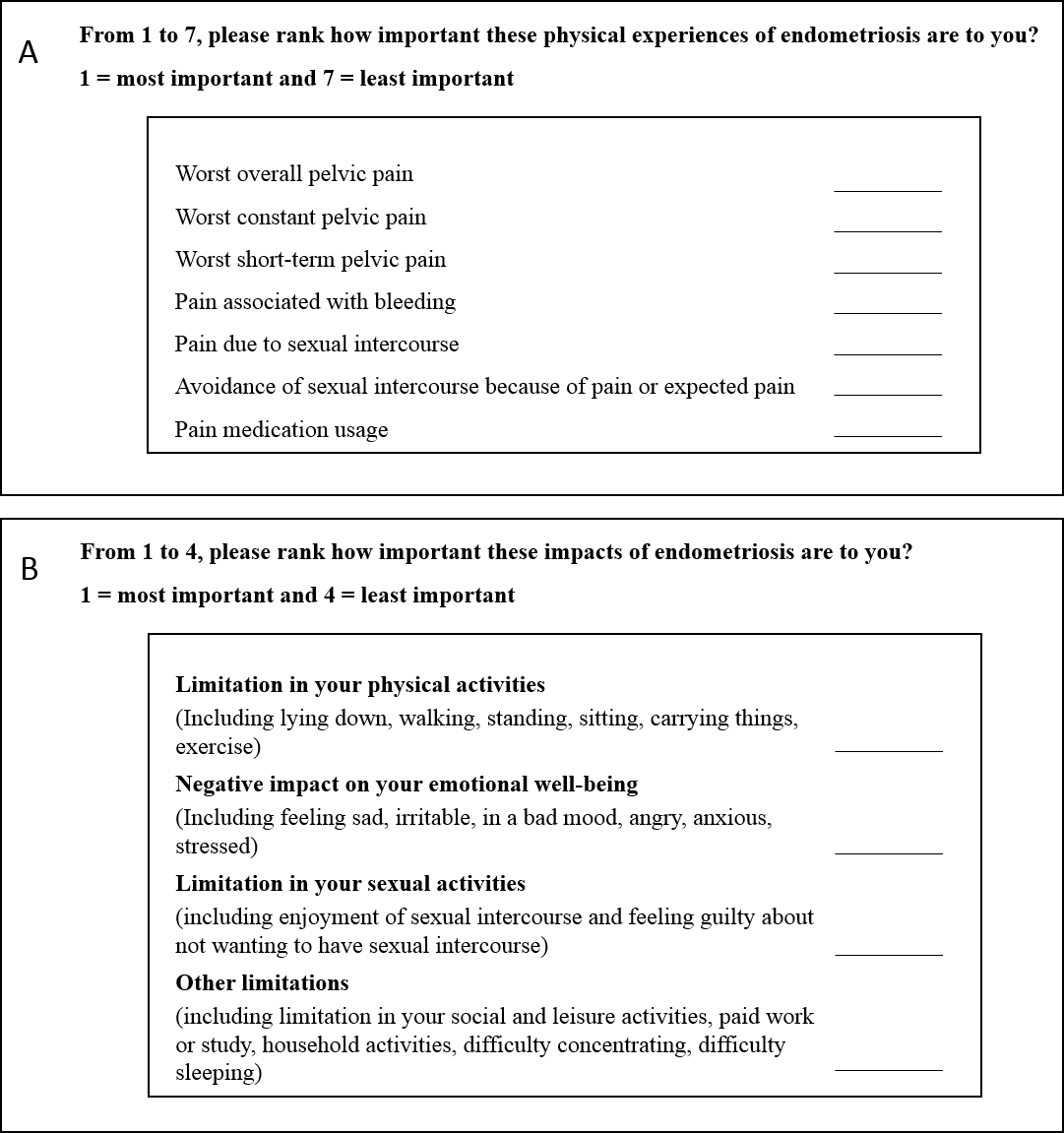


Abbreviations: *EIS* Endometriosis Impact Scale; *ESD* Endometriosis Symptom Diary

**Additional Figure 3** 28-day scoring option calendars to explore algorithms for ESD “worst pelvic pain” 0–10 NRS.


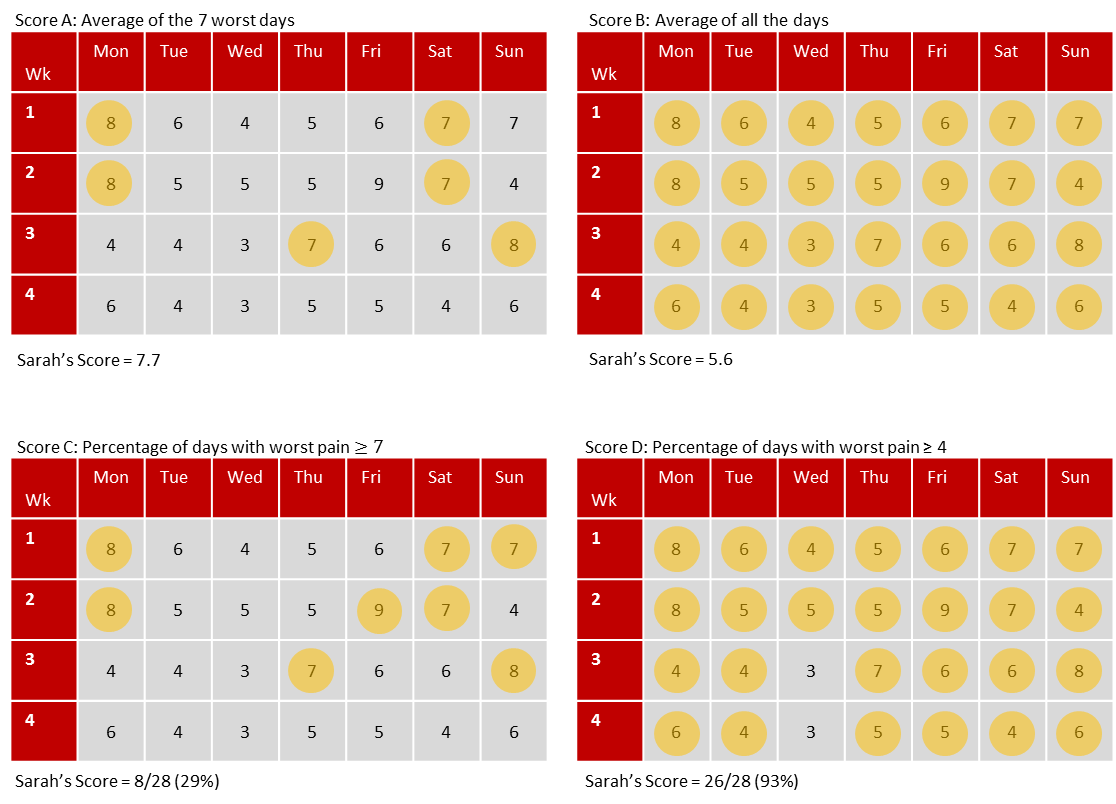


Abbreviations: *ESD* endometriosis symptom diary; *NRS* numeric rating scale.

Additional Figure 4 Blank numerical rating score (NRS) for cut-off score activities


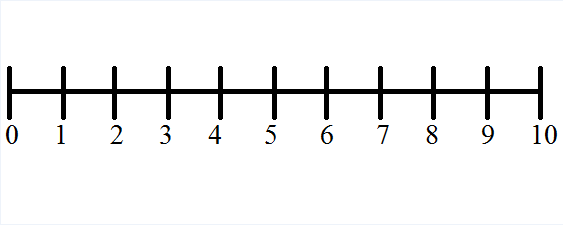


**Additional Figure 5** Example of a depiction of a score change on the endometriosis symptom diary (ESD) “worst pelvic pain” to facilitate discussion of meaningful change.


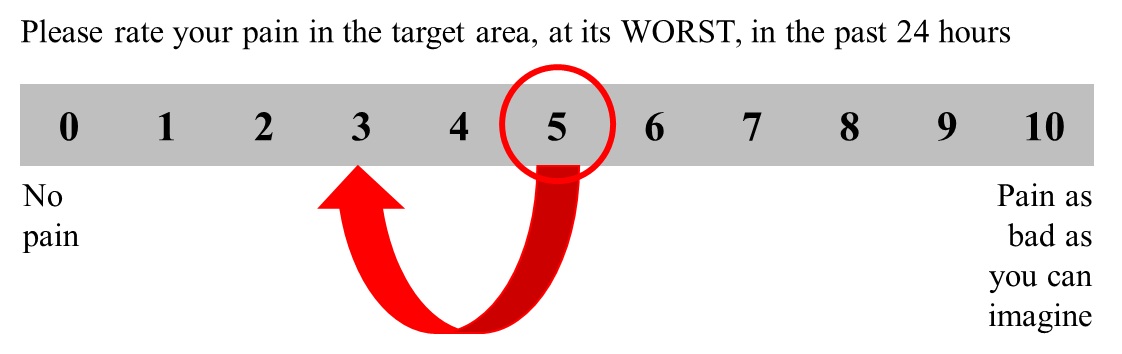

Supplement: Supplementary file 1 — Additional file 1. Examples of completed ranking tasks and material used for cognitive exploration tasks. [file 12955_2020_1579_MOESM1_ESM.docx]
